# “Adhesions after abdominal, pelvic and intra-uterine surgery and their prevention”

**DOI:** 10.1007/s10397-012-0762-4

**Published:** 2012-08-16

**Authors:** Markus Wallwiener, Hans Brölmann, Philippe Robert Koninckx, Per Lundorff, Adrian M. Lower, Arnaud Wattiez, Michal Mara, Rudy Leon De Wilde, The Anti-adhesions in Gynecology Expert Panel (ANGEL)

**Affiliations:** 1 Department of Obstetrics and Gynaecology, University of Heidelberg, Vosstr. 9, 69115 Heidelberg, Germany; 2 VU University, Amsterdam, The Netherlands; 3 University Hospital Gasthuisberg, Katholieke Universiteit, Leuven, Belgium; 4 Viborg Hospital, Viborg, Denmark; 5 The Womens’ Health Partnership Ltd, London, UK; 6 Hôpital de Hautepierre, Strasbourg, France; 7 Charles University, Prague, Czech Republic; 8 Pius-Hospital Frauenklinik, Oldenburg, Germany

**Keywords:** Adhesions, Adhesiolysis, Adhesion prevention, Patient education

## Abstract

We here present the full text of a patient leaflet we have designed, and routinely use, to provide preoperative education about adhesions to patients undergoing open or laparoscopic gynaecological surgery. The leaflet presents appropriate, patient-orientated information on the nature of adhesions, their causes and the health risks they may involve as well as on adhesiolysis and modern methods of adhesion prevention. As adhesion formation is not specific to gynaecological surgery, the leaflet can also be adapted for the purposes of general abdominal surgery.

In the following, we present the full text of the leaflet. Abdominal and pelvic surgical procedures often result in adhesions that can cause patients to suffer chronic pain, nausea, vomiting, female infertility, constipation or small bowel obstruction [1] even after many years.

This Information Leaflet is intended to provide basic information about prevention or reduction of postsurgical adhesion formation in the abdominal pelvic area.

Adhesions are among the most common side effects after abdominal surgery. Despite good surgical practice, a residual risk remains.

Adhesions are fibrous strands/bands of tissue that form during the wound healing process and bind adjacent organs and tissues together that normally are separate. Adhesions can form as a result of surgery involving the peritoneum or abdominal and pelvic organs. Injury to the peritoneum is unavoidable during surgical interventions in the abdominal cavity but can also develop as a result of inflammation or mechanical injury.

There are different types of invasive gynaecological procedures, which potentially cause adhesions. Patients undergoing tubal, ovarian or uterine procedures are at high risk. Open surgical interventions in the pelvic area, such as surgical treatment of endometriosis, tumour removal and bowel surgery, are associated with an increased risk due to the trauma caused to the abdominal membrane (peritoneum). Adhesions may also form after laparoscopic or hysteroscopic removal of fibroids.

Not all adhesions impair health or well-being, but they are the most common cause of difficulty becoming pregnant (reduced fertility), chronic lower abdominal pain as well as constipation and may even cause life-threatening bowel obstruction (ileus). Such adhesions can reduce quality of life for patients [2]. In such cases, surgical treatment of adhesions is often required.

Currently, the only way to relieve an adhesion is through surgical separation (adhesiolysis); however, reformation occurs postoperatively in most patients (on average 85 %) regardless of what adhesiolysis/operation technique is used [1], hence the importance of attempting to prevent initial adhesions from forming.

The risk of adhesion formation can be reduced by good surgical practice such as avoiding bleeding, minimising tissue handling, reducing infection and contamination risk, reducing drying of tissues, limited use of cautery, limited use of sutures, avoiding foreign bodies and use of starch free gloves [3]. Your surgeon will do his/her best to reduce the risk of adhesion formation and thus avoid such “triggers”. However, these efforts will not always be enough to prevent adhesions entirely.

The risk of adhesion formation after abdominal or pelvic surgery can be lowered further through the use of certain products. Some products reduce the formation of adhesions by affecting the biological processes involved in adhesion development. Others form mechanical barriers between the organs and tissues, thus providing a temporary separation after surgery.

Mechanical separation of organs is achieved by using special fluids, films or gels applied to the area at risk of forming adhesions. During the healing period, those products prevent the wound areas from sticking to adjacent tissues. Most products are then reabsorbed after a few days.

Severity and type of symptoms may influence the choice of prophylactic treatment. The most suitable product for you depends on the surgical procedure and expected risk of adhesion formation. Your surgeon may suggest which adhesion-reducing products could be used in your planned surgical procedure, in addition to good surgical practice [4, 5].
